# Quickly evolving near-infrared photoimmunotherapy provides multifaceted approach to modern cancer treatment

**DOI:** 10.1002/VIW.20200110

**Published:** 2021-08-27

**Authors:** Hailey Monaco, Shinya Yokomizo, Hak Soo Choi, Satoshi Kashiwagi

**Affiliations:** 1 Gordon Center for Medical Imaging, Department of Radiology, Massachusetts General Hospital and Harvard Medical School, Boston, Massachusetts, USA; 2 Department of Radiological Sciences, Tokyo Metropolitan University, Arakawa, Tokyo, Japan

**Keywords:** cancer immunotherapy, immunogenic cell death, near-infrared photoimmunotherapy, photodynamic therapy, photothermal therapy

## Abstract

Among modalities of cancer immunotherapy, near-infrared photoimmunotherapy (NIR-PIT) has reached significant preclinical and clinical stages and quickly evolved over the last 5 years. NIR-PIT uses deep-penetrable NIR light to induce physicochemical changes in the antibody–photosensitizer conjugate (APC), leading to resultant necrosis and immunogenic cell death (ICD) of the cancer cells. Alternatively, other types of photomedicine use photosensitizers to convert absorbed light energy either into reactive oxygen species for photodynamic therapy (PDT) or into heat for photothermal therapy (PTT). ICD is a unique and relevant outcome of NIR-PIT because it induces long-lasting antitumor host immunity, which overcomes the immunosuppressive network of cancer. Due to its high specificity and durable antitumor effects, NIR-PIT is now considered a promising cancer therapy, and optimized NIR-PIT is readily expanding its applicability to many different types of cancer. Along with the traditional method of NIR-PIT, new avenues in its realm of treatment are currently being explored, such as the targeting of other immunosuppressive elements, delivery of NIR light through a catheter, real-time imaging for tumor detection, and the use of tumor-seeking small molecules for improved efficacy and safety. In addition, its effect on hyperpermeability has opened a door for a wide array of combination therapies with other modalities. This review summarizes the recent findings in clinical and preclinical studies of NIR-induced photomedicine and its future significance in the field of cancer research.

## 1 | INTRODUCTION

For the past 100 years, the main contenders in cancer therapy have been chemotherapy, surgery, and radiotherapy.^1^ Although these modalities have proven to decrease tumor burden and may lead to remission, they generally do not provide a predictable method for achieving long-term remission, and in some cases, result in worse cancer progression due to their nonspecific targeting nature and subsequent suppression of the host immune system. Depending on the type of drugs used, dosage, and treatment frequency, radiation therapy and chemotherapy can compromise the innate and adaptive immunity in cancer patients, which would normally play a critical role in fighting pathogenic threats.^2^ Patients with late-stage metastatic cancers often face bleak treatment options as primary tumor cells can metastasize through the blood stream or lymphatics to other sites of the body, causing immune system and vital organ dysfunction. Although the modern era of cancer research has been underway for more than two centuries and the overall cancer death rate has undoubtedly decreased year-by-year, there are still many types of cancer that remain extremely prevalent and aggressive, such as lung, prostate, breast, colorectal, and stomach cancer.^3^ For example, in 2020 alone, an estimated 1.8 million people died from lung cancer of the 2.2 million people diagnosed globally, which demonstrates an alarmingly low 18% survival rate for one of the most prevalent types of cancer.^3^ Similar statistics accompany other types of cancer, such as the ones mentioned above, which proves that there is a clear need for a compatible cancer treatment to emerge that addresses these low survival rates and improves prognosis overall.

The main downfalls of current cancer treatments are associated with their tendency to indirectly damage normal tissue, leaving the host immune system dysfunctional and at greater disadvantage than it was prior to treatment. A treatment avenue that has been investigated within more recent years is cancer immunotherapy, which incorporates the host’s antibodies, cytokines, and dendritic cells (DCs) to activate a response against tumor cells. Normally, tumor cells would escape the immune system due to the accumulation of immature myeloid cells (IMC) in the tumor microenvironment (TME), which subsequently induces strong immunosuppressive effects.^4^ IMCs differentiate into granulocytes, macrophages, and DCs under normal cellular conditions, but cannot do so in the TME due to chronic inflammatory stress.^5^ In particular, immune checkpoint inhibitors, including cytotoxic T-lymphocyte-associated protein 4 (CTLA-4) and programmed cell death protein 1 (PD-1)/programmed cell death 1 ligand 1 (PD-L1) inhibitors, which interfere with suppressive signals from the TME, have demonstrated remarkable increases in long-term progression-free survival (PFS) of late-stage melanoma and revolutionized the treatment of various types of cancer.^6^ These recent results in immunotherapy indicate that augmentation of anticancer immunity has the potential to eradicate cancer. Although immunotherapy presents a unique and effective mechanism of potentially treating cancer through long-term antitumor immunity, it can also present serious dangers to the host system, such as autoimmunity and nonspecific inflammation.^7^ In addition, the current immunotherapy methods have only benefited a small fraction of patients. For example, a recent study in non-small cell lung cancer patients undergoing immunotherapy targeting PD-L1 reported that 82% of participants had died by the 5-year data cutoff point, though patients with high PD-L1 expression and naïve to previous cancer treatment responded better and had longer overall survival (OS) when treated with immunotherapy.^8^ Therefore, although current immunotherapy trends positively contribute to OS rate in advanced cancers, there is still much room for improvement to achieve convincing long-term PFS rate in a wide range of aggressive cancer types.

In short, all of the above cancer treatments, which are currently used in clinical settings—chemotherapy, surgery, radiotherapy, and immunotherapy—present a unique, but only semi-successful way to mediate cancer progression. New therapeutic modalities therefore need to be designed to specifically avoid collateral damage on the normal tissue and immune system, and these new modalities could be combined with classic approaches to yield exceptional outcomes.

## 2 | CANCER PHOTOMEDICINE

### 2.1 | Photodynamic therapy

**FIGURE 1:**
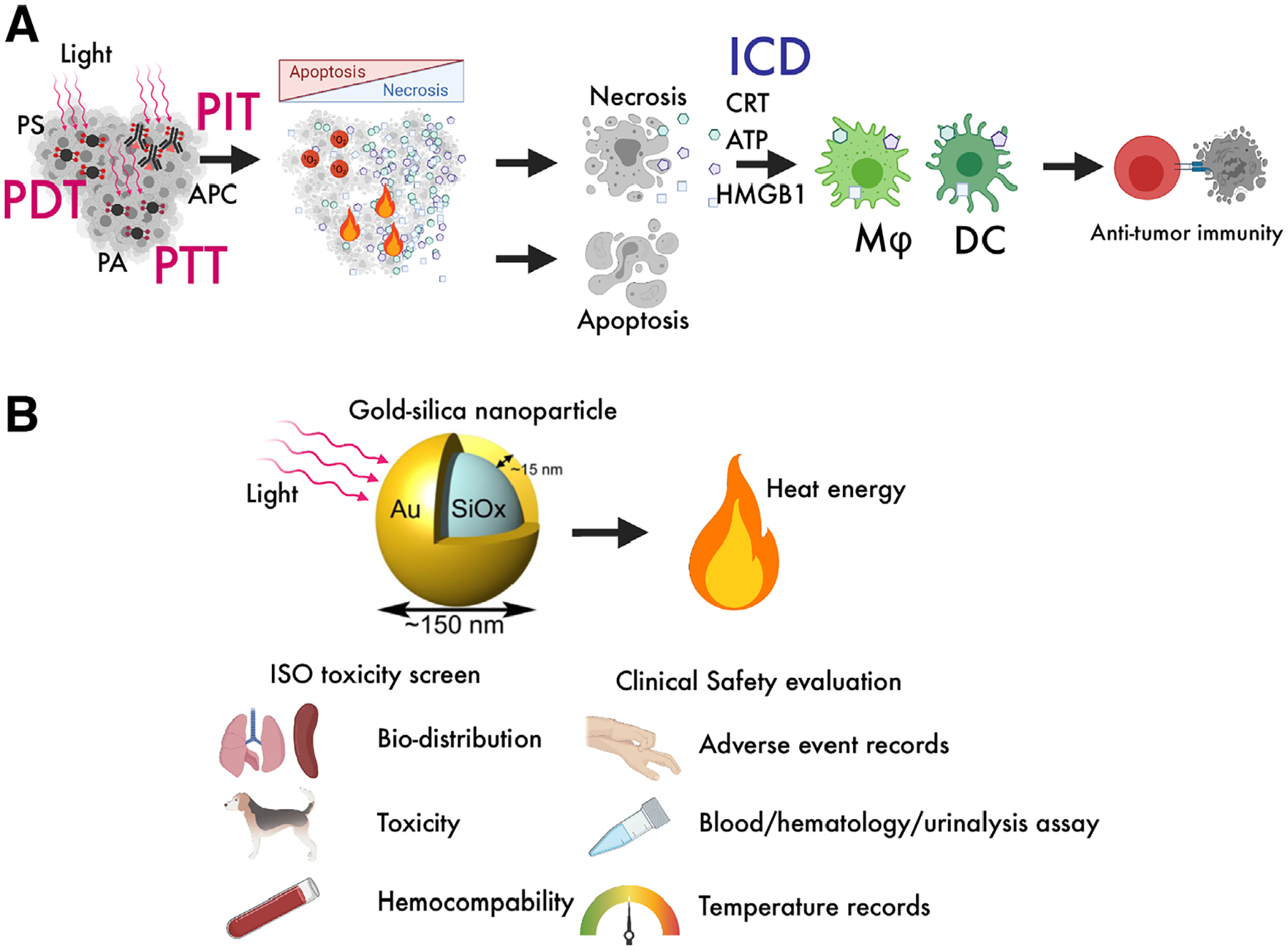
(A) Near-infrared photoimmunotherapy (NIR-PIT) and other modalities. NIR-PIT shows remarkable therapeutic effects inducing long-lasting antitumor host immunity via inducing immunogenic cell death. NIR-PIT evokes robust anticancer immunity via release of damage-associated molecular pattern. (B) Schematics of AuroLase clinical study. AuroLase is a photoabsorber gold-silica nanoparticle and shows PTT effect upon NIR-irradiation. The efficacy and safety of this approach has been well validated and more clinical trials for PTT with AuroLase are ongoing for various types of cancer. Modified from Paithankar et al.^40^ 2015 with permission from Elsevier B.V.

Photodynamic therapy (PDT) has gained widespread attention as a targeted photomedicine technique in modern cancer research, which involves the activation of a photosensitizer (PS) through light-based therapy (Figure 1A).^9^ PDT induces photosensitized oxidation of specific molecules, most notably triggering reactive oxygen species (ROS) to cause cell death in cancer cells by damaging their DNA, membranes, proteins, and certain surrounding normal cells.^10^ This approach has proved effective as a cancer treatment in both preclinical and clinical settings. In addition to the inherent targetability of a PS, focused delivery of light to the tumor adds another layer of specificity to avoid significant normal cell damage. Through this mechanism, PDT predominantly induces apoptosis of cancer cells,^11^ which could negatively result in tolerogenic cell death and tolerance to these cells.^12^ It was also found that PDT can also sometimes induce necrosis depending on various treatment conditions, and multiple studies have shown that necrosis was more likely to occur in PSs that localized in cell membranes or lysosomes, compared to the mitochondria, which was hypothesized to lead to apoptosis.^13^ Apoptosis is the process of programmed cell death for regulation and autoimmune discrimination, which is immunologically silent due to the fact that it also occurs under normal physiological conditions^14^ and does not produce a long-lasting immune response that would alert the host to mobilize against the tumor cells.^15^ Furthermore, due to the limitation of laser penetration into tissues, the majority of PDT studies involve the treatment of superficial lesions of skin or luminal organs. Numerous clinical studies have been conducted for PDT to date.^10^ Although lack of immune response in PDT due to silent apoptosis and limitations of laser penetration are drawbacks, these clinical studies in patients with recurrent breast cancer,^16^ malignant biliary obstruction,^17^ cholangiocarcinoma,^18^ and basal cell carcinoma^19^ have all seen clinical benefits in Phase 1, 2, or 3 trials. Based on these results, a number of trials using PDT alone or in combination with other therapies are ongoing for different types of cancer including basal carcinoma, non-melanoma skin cancer, anal cancer, brain tumors, glioblastoma, neurofibroma, head and neck cancer, oral cancer, breast cancer, lung cancer, mesothelioma, esophageal cancer, gastric cancer, pancreatic cancer, cholangiocarcinoma, bladder cancer, prostate cancer, and cervical cancers as well as for cervical dysplasia and colorectal cancer.^10,20^ PDT demonstrates the efficacy of photomedicine to treat cancer and would be a good candidate for combination treatment with immunotherapy to create both an immediate and sustained antitumor response.

Recent studies in the combination of immunostimulants with PDT have indeed been reported to enhance antitumor immunity.^21^ In the treatment of 4T1 breast cancer, immunocompetent BALB/c mouse models underwent PDT and immunotherapy through a PS of verteporfin combined with peritumoral injection of immunologic adjuvant, CpG-ODN. This resulted in direct tumor cell death from verteporfin with NIR light irradiation and DC maturation and phagocytosis in TME by CpG-activated immune response. Compared to groups that only received PDT treatment or only immunologic adjuvant treatment, PDT and CpG combination treatment resulted in the greatest reduction in tumor size and longest survival time. In a similar preclinical PDT-immunotherapy combination study in a colorectal cancer model, targeted upconversion nanoparticles (chlorin e6 PS) and immunologic adjuvant, imiquimod (R837) targeting Toll-like receptor 7 (TLR7) were combined with immune checkpoint blockade therapy of CTLA-4.^22^ The results of this study showed direct killing of tumors targeted by the nanoparticles and NIR laser, the development of antitumor immunities established by the immunologic adjuvant, and continued immune checkpoint blockade therapy and inhibition of growth of distant tumors. Hwang et al. also demonstrated synergy between PDT and tumor-specific peptide vaccination adjuvanted with TLR5 agonist flagellin in a mouse model of melanoma.^23^ In this study, the combination therapy induced a robust increase in tumor infiltrating CD8^+^ T cells and migratory CXCL10-secreting CD103^+^ cross-presenting DCs, resulting in systemic antitumor immune responses for both local and abscopal tumor control. These preclinical studies suggest that PDT could be successfully combined with immunotherapy to achieve long-term cancer remission.

### 2.2 | Photothermal therapy

Alternatively, photothermal therapy (PTT), which converts light energy into heat to induce direct cancer cell death at the target site, is also being explored and has proven effective in preclinical and clinical settings for cancer photomedicine (Figure 1A).^24^ PTT can be combined with a photoabsorber such as the commonly used fluorescence dye, indocyanine green (ICG), to facilitate heat generation.^25^ As for targeted, therapeutic agents, recent PTT studies in preclinical settings have mainly used either noble metal, carbon-based, metal compound, or organic nanomaterials.^26^ PTT treatment alone and in combination with chemotherapy or immunostimulants has proven successful in various preclinical studies targeting breast cancer^27^ and hepatocarcinoma^28^ to significantly reduce primary tumor size and multidrug resistance through hyperthermia. Recently, more combination PTT-immunotherapeutic strategies have emerged, with the goal of simultaneously killing primary tumor cells, while boosting long-term antitumor immunity in distant and metastatic tumors.^29^ Early studies of PTT, which led to the initial concept of photoimmunotherapy (PIT), were first proposed by Dr. Wei R. Chen and colleagues in 1997,^30^ which later led to the discovery that PTT is mediated by photothermal and robust anticancer immune responses.^31^ For example, Zhou et al. exhibited that NIR laser treatment increased the temperature and delayed growth of tumors in syngeneic mouse models of pancreatic cancer and melanoma.^32^ In this approach, tissue heat generation led to tumor cell death and release of tumor-associated antigens (TAAs), which acted as a mechanism of autologous cancer vaccination. The heat generation also led to release of damage-associated molecular patterns (DAMPs) including calreticulin (CRT), heat shock proteins (HSPs), high-mobility group box 1 (HMGB1), and adenosine triphosphate (ATP), thus inducing immunogenic cell death (ICD).^33^ In response to these stimuli, DCs increased their activation marker expression of MHCII and CD8^+^ and induced further recruitment of DCs in TME. These activated DCs migrate to the lymphoid tissue and augment tumor-specific cytotoxic T-lymphocyte response in collaboration with adjuvant. The primed CD8^+^ T cells then infiltrate TME and perform immunosurveillance. This immune response can be further augmented via immunologic adjuvant implementation. Other studies consistently demonstrated that NIR irradiation along with topical imiquimod application induced release of DAMPs suppressing tumor growth in breast and skin cancer models in mice.^34^ Following these in vivo results in animal models, the combination of PTT and immunologic adjuvants for refractory cutaneous warts^35^ and immune checkpoint inhibitors for melanoma^36^ was tested and proved effective in clinical studies.

In clinical trials of PTT, photoabsorber-conjugated gold-silica nanoparticles, known as AuroLase were used to treat tumors for advanced melanoma (Figure 1B). Upon NIR-irradiation, AuroLase converted the light to heat energy, which induced highly localized hyperthermia, cell death, and subsequent tumor remission.^37^ By manipulating the inner and outer radius of the gold shell within the nanoparticle, gold-silica nanoparticles have proven successful in absorbing varying wavelengths.^38^ Among 15 male prostate cancer patients treated with AuroLase intravenous injection and then exposed to NIR light through optical fibers inserted in the solid tumors, 13 were cancer free in their 1-year follow-up biopsies, which showed the promising outcomes in future PTT for cancer.^39^ More clinical trials using AutoLase have also taken place, in both metastatic lung cancer and head and neck cancer, but neither were able to sufficiently measure the effect in targeted tumors (NCT01679470, NCT00848042), and therefore more clinical trials are necessary to confirm the efficacy of this form of PTT. In addition, in future clinical trials for PTT, it would be beneficial to monitor its effects on the anticancer immune responses, which has not been monitored thus far, in order to determine the potential efficacy of combining PTT with immunotherapy to achieve long-term cancer remission

### 2.3 | Photoimmunotherapy

The history of current cancer treatments and novel discoveries in photomedicine has opened the door for a new modality to emerge that directly kills cancer cells, possesses high specificity for tumor tissue, and induces long-term PFS by decreasing the chances of recurrence through augentation of the immune system via ICD. This solution comes in the form of another variation of photomedicine, near-infrared photoimmunotherapy (NIR-PIT) (Figure 1A). NIR-PIT has been proposed and established by Dr. Hisataka Kobayashi and colleagues at the National Institutes of Health. NIR-PIT is a modern cancer therapy that combines targeted immunotherapy and photochemistry-based therapy, and was recently approved for treatment of unresectable head and neck squamous cancer in Japan.^41^ This technology has reached clinical stages at an unprecedented speed worldwide with solid safety and efficacy data. It has been in Phase 1/2/3 clinical trials globally since 2015.^41^

### 2.4 | Photobiomodulation

There is solid evidence for diverse biological effects resulting from treatment with NIR light at low power including tissue regeneration, analgesia, and reduction of the inflammation.^42–44^ These effects are broadly defined as photobiomodulation (PBM)^43^ and consist of another important photomedicine which parallels PDT, PTT, and PIT. PBM in the context of cancer therapy typically involves no prior chemical or small-molecule injection in the host to the therapy and uses endogenous photoabsorbers.^45,46^ Because some studies showed that PBM stimulated the growth of cancer cells in vitro, it continues to be controversial whether PBM to tumor sites overall stimulates or suppresses cancer growth.^47^ In fact, several studies showed that 650–660 nm laser treatments increased the tumor size in mouse models of subcutaneous melanoma^48^ and anaplastic thyroid cancer.^49^ However, dominant contradictory evidence suggests that PBM can suppress tumor progression via multiple mechanisms. Within the tumor, glycolysis, which consumes less oxygen than oxidative phosphorylation, is the main form of energy production. This is called the Warburg effect, and is a key difference between cancer and normal cells in the context of PBM.^50^ In normal cells, PBM stimulates mitochondrial retrograde signaling and increases ATP production via activation of cytochrome c oxidase (COX) and subsequent generation of ROS.^44^ In contrast, cellular ATP is limited in cancer cells, and an increase in ATP with PBM may force the cancer cells to exaggerate their precarious metabolic state responding to pro-apoptotic stimuli, resulting in cell death.^50^

Consistently, Khan et al. recently demonstrated the phototoxic effect of PBM using NIR laser with no genotoxic or mutagenic capacity. In this study, excessive doses of PBM promoted cell death through autophagy and apoptosis through the concerted action of ROS and heat via expression of ATL-4 and HSP70.^51^ In addition, high-fluence low-power NIR laser exposures (HF-LPLI) at 632 nm^52^ induced tumor regression of EMT6 breast tumors in mice.^53^ Interestingly, this effect was not observed in rho-zero EMT6 tumors that lack functional mitochondria, which suggests mitochondrial involvement in PBM. PBM is also known to affect specific signaling via receptors including opsins, aryl hydrocarbons and growth factors including TGF-*β*1, which has potential to significantly impact the fate of tumor cells.^45^

Although the exact mechanisms are largely unclear, PBM appears to stimulate the immune system. In the HF-LPLI study,^53^ as EMT6 tumors are highly immunogenic, long-term immunological memory was observed in the host. In addition, Ottaviani et al. demonstrated the PBM in a mouse model of melanoma with 660–970 nm NIR laser delayed tumor growth and increased the recruitment of T cells and DCs.^54^ Similarly, Petrellis et al. showed that PBM on the tumor site of Walker sarcoma model rats using 660 nm laser increased expression of IL-1*β*, COX-2, and iNOS but decreased IL-6, IL-10, and TNF-*α*, suggesting the immunomodulatory effects of PBM.^55^ These results indicate that the main mechanisms of PBM could involve stimulation of the immune system.

There are two clinical trials demonstrating increased survival in cancer patients who received PBM. PBM using 660 nm laser was applied on oral mucositis areas in cancer patients and significantly improved PFS. It also resulted in a tendency for better OS, although it is not clear if PBM exerted a direct anticancer effect.^56^ Consistently, PBM using 904 nm laser treatment of the skin in cancer patients significantly improved PFS and quality of life (QLI) with no dose-limiting toxicity.^57^ For cancer survivors in this study, an initial increase in TNF-*α* followed by a decrease was observed in the peripheral blood, although a progressive increase in TNF-*α* and sIL-2R in nonsurvivors was noted, which demonstrates possible immunomodulatory effects of PBM. Given the broad range of PBM effects and its precise impact on the tumor progression, further investigation on the mechanisms of action of PBM in the context of cancer therapy is warranted. Overall, PBM is an important photomedicine to consider as a parallel field to NIR-PIT.

## 3 | MECHANISM OF NIR-PIT

The mechanism of NIR-PIT involves a monoclonal antibody (mAb) bound to a photoactivating silicon phthalocyanine dye, IRDye700DX (IR700), which is injected into the host systemically.^41^

**FIGURE 2:**
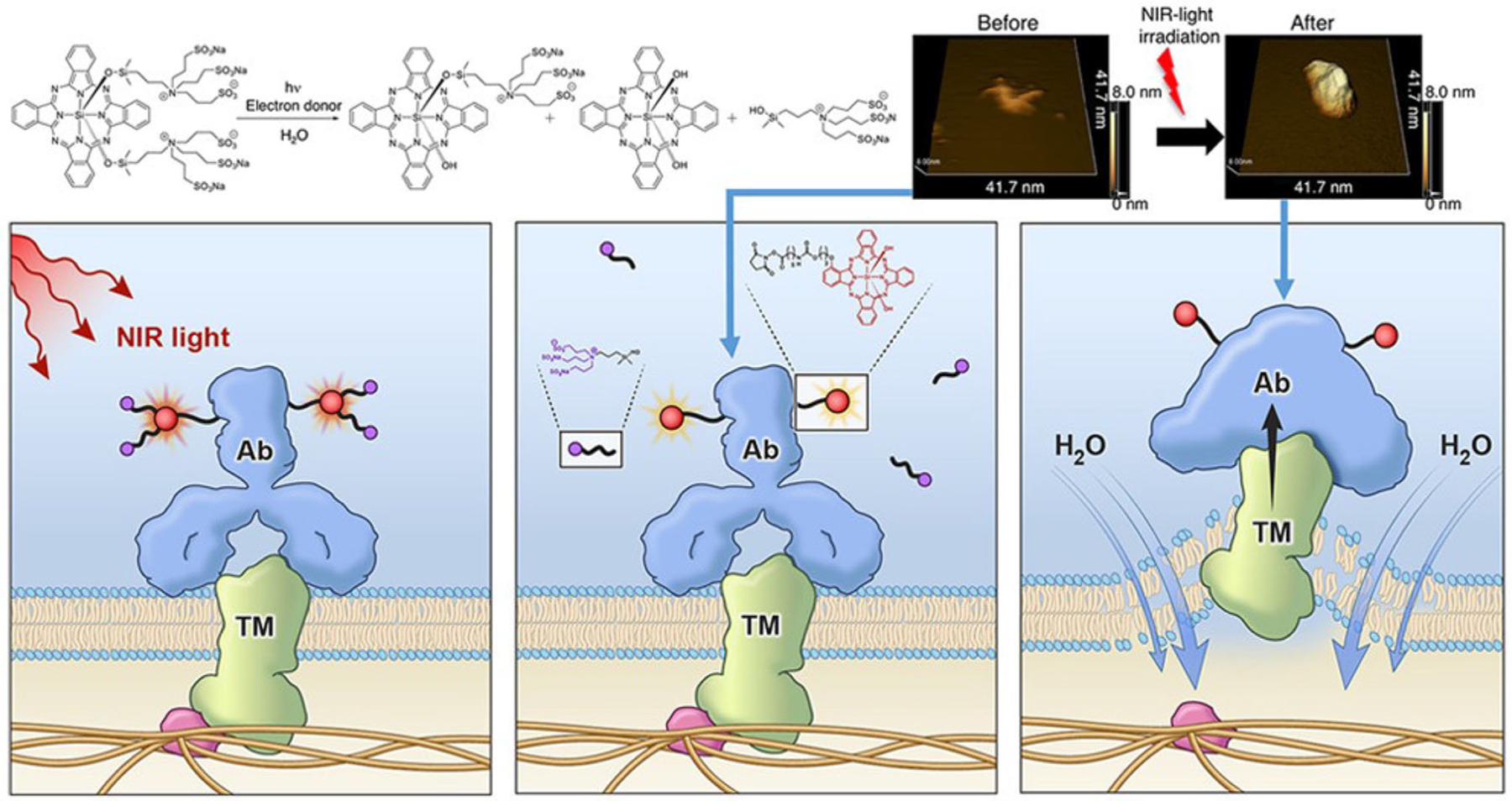
Mechanism of action of near-infrared photoimmunotherapy (NIR-PIT). A monoclonal antibody–photoabsorber (IR700) complex (APC) binds tumor antigen on the cell surface. NIR light irradiation triggers release of ligands form APC and induces the changes in solubility, which produces physical stress on and disrupt integrity of the cell membrane, and then the water outside of the cell enters the cell to ultimately burst the cell. Ab; antibody, TM; tumor marker (antigen). Reprinted from Kobayashi et al.^41^ 2021 with permission from the American Chemical Society

IR700 is the only PS that has proven consistently successful in NIR-PIT studies thus far. Once the antibody conjugate is administered systemically, it travels and binds to its target biomarker antigen, which is overexpressed on the surface of cancer cells, and NIR light is exposed to the area of the tumor tissue to activate the PS. NIR-PIT light exposure of 690 nm triggers photoinduced physicochemical changes in the bound antibody–photoabsorber complex (APC) and causes the cancer cell membrane integrity to degrade in a necrotic manner. Water begins to flood into the cancer cell from its surroundings, until the cell eventually swells and bursts, releasing its antigens and genetic materials into the environment (Figure 2). Importantly, these changes upon NIR-PIT uniquely induce stress responses in dying cells, which simultaneously stimulates host immune response.^41,58^ Due to NIR-PIT’s ability to highly specifically bind to cancer surface cell antigens, it causes rapid induction of cell killing at the tumor site and activation of systemic antitumor immunity, while leaving surrounding normal tissue healthy and keeping the immune system intact. For these reasons, NIR-PIT presents a feasible therapeutic avenue for photomedicine cancer treatment to achieve long-term remission.

## 4 | CELL DEATH FOLLOWING NIR-PIT

### 4.1 | Immunogenic cell death

**FIGURE 3:**
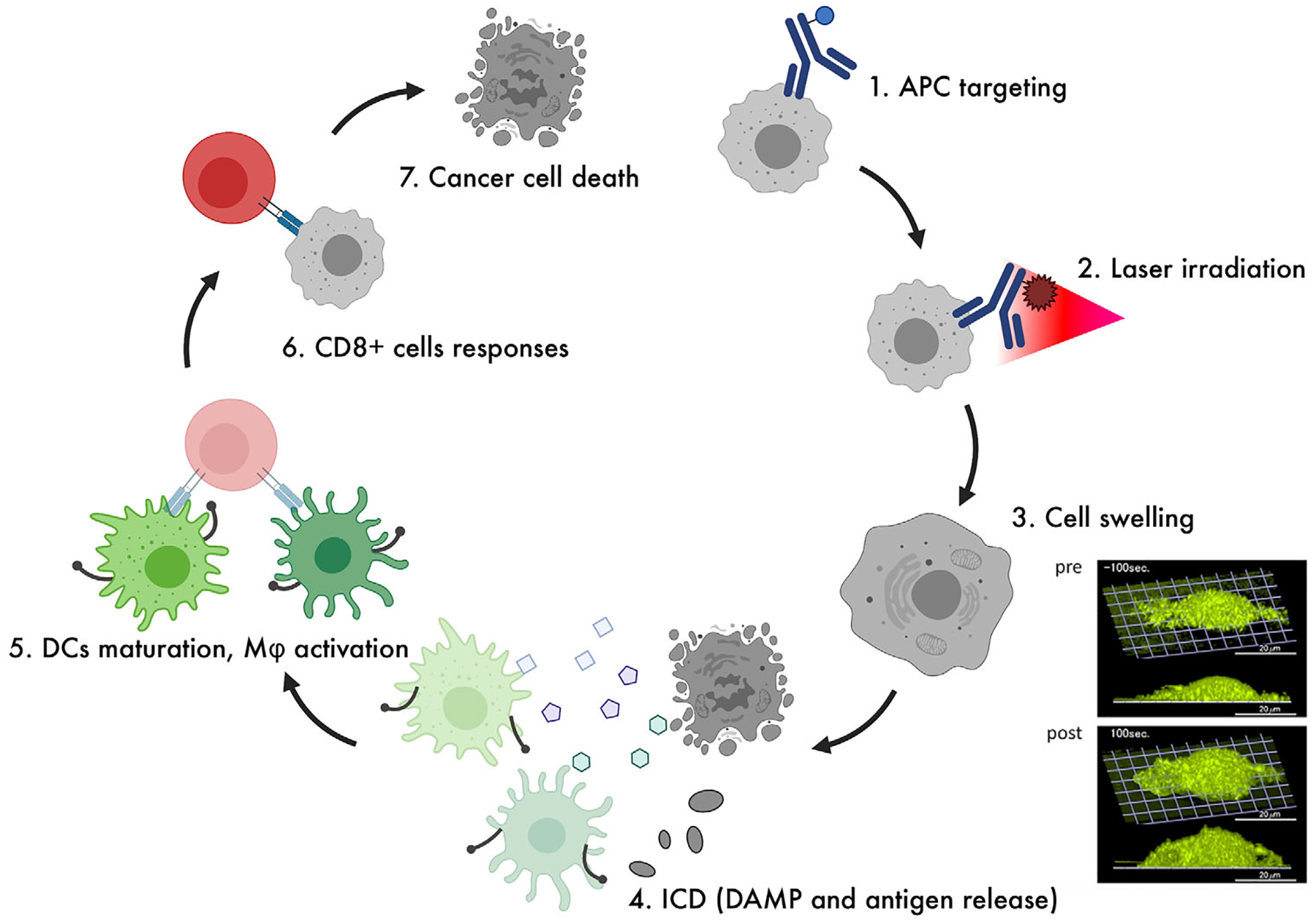
Immunological mechanisms of action of NIR-PIT. NIR irradiation induces rapid stress response and necrosis in cancer cells through physicochemical changes in the APC. Rapid cell death leads to the release of cancer-specific antigens, and activation of the immune system through maturation of DCs and priming of CD8^+^ cells. Modified from Ogawa et al.^58^ 2017 with permission from the Impact Journals, LLC

The intriguing part of NIR-PIT is that it invokes two different antitumor responses in the host, which work together to increase the success of this treatment. The first antitumor response is the direct killing of the cancer cell by cytotoxic effect triggered by irradiation of the APC, which is common in all phototherapies, but the second response, which is ICD, is unique to NIR-PIT. This stimulates long-term immune responses to the cancer-specific antigens released from the dying cells. Similar to PTT, ICD induced by NIR-PIT relies on immunogenic signals including DAMPs such as calreticulin, ATP, HMGB1, Hsp70, and Hsp90.^58^ However, laser irradiation in NIR-PIT generally does not result in extensive heat generation, which is distinct from PTT.^41^ Upon bursting, the cell releases its TAAs and DAMPs in the surrounding environment, which promotes maturation of immature DCs and subsequent TAA capture by DCs.^59,60^ These mature DCs prime naive T cells to become cancer-specific CD8^+^ T cells in the lymphoid tissue via cross-presentation of TAAs on MHC I, augmenting host antitumor immune response (Figure 3). The primed CD8^+^ T cells can then perform immunosurveillance throughout the body to eliminate primary or metastatic cancer expressing the same target biomarker antigens. The increasing levels of dying cells leads to more release of TAAs and immunogenic factors, which amplifies the immune response by broadening target antigens over time (antigen spreading).^59,61^ Thus, ICD is an important component of NIR-PIT because it leads to long-term immune effects even after initial rapid cell death. This feature differentiates NIR-PIT from conventional cancer treatment methods that do not typically initiate robust immune response, but rather mediate immune tolerance to the cancer, allowing for tumor progression.^62^ At this moment, there is no study that directly analyzes modes of photomedicine to compare which modality induces the most robust ICD, which warrants further investigation.

### 4.2 | Transcription factor 4 (ATF-4)-mediated phototoxicity

Although NIR laser treatment including PBM is largely viewed as cytoprotective, evidence suggests that PBM can suppress tumor progression. Importantly, recent studies from Dr. Praveen R. Arany and his colleagues revealed a new concept of cell death with phototoxic effect of PBM. This study demonstrated that ATF-4 induced by NIR laser treatment was a contributing factor to the cell death mechanism of PBM.^51^ NIR laser with no genotoxic or mutagenic capacity was able to promote cell death in a concerted action of ROS and heat via expression of ATF-4 and HSP70.^51^ At the correct dosage, the endoplasmic reticulum (ER) mediated by ATF-4 could lead to autophagy and apoptosis. Upon NIR exposure, this mechanism might contribute to ICD and a robust antitumor immune response. More investigations are necessary to determine the possible role of ATF-4-mediated phototoxicity in NIR-PIT, which would lead to greater understanding of the thresholds and ideal dosage of NIR light for treatment.

## 5 | NIR-PIT TARGETING TUMOR BIOMARKERS IN ANIMAL MODELS

The efficacy of NIR-PIT has been demonstrated in various tumor models both in vitro and in vivo. In an in vitro study of mycosis fungoides (MF), which is a type of low-grade T-cell lymphoma, cutaneous lymphocyte antigen (CLA) is targeted by an anti-CLA antibody conjugated with IR700, which successfully induced substantial increase in MF cell death.^63^ Loss of viability of cells upon NIR irradiation was observed in this study. In another in vitro study of head and neck cancer cells, the oncogenic epidermal growth factor receptor (EGFR)^64^ was targeted by anti-EGFR antibody, cetuximab, conjugated with IR700, which similarly induced cell death due to the rupture of the cell membrane of cancer cells and elicited the subsequent activation of co-cultured DCs.^58^ In a study of an in vivo xenograft model of human bladder cancer, targeting CD47, which is an innate immune checkpoint found predominantly on bladder cancer cells, resulted in increased cytotoxicity in cancer cells denoting an innate response upon NIR-PIT using anti-CD47 antibody.^65^ Consistent with this in vivo data, in vitro analysis demonstrated that treatment of cancer cells with NIR-PIT resulted in increased phagocytosis activity of co-cultured macrophages.^65^ It is worth noting that cytotoxicity had a direct relationship with the dose of NIR light irradiation, based on comparison of power ranging from 1 to 40 J/cm^2^. Interestingly, the fluorescence intensity of treated tumors notably decreased in the treatment group by 72 h, suggesting that the treatment effect can be monitored by changes in fluorescence intensity. Overall, NIR-PIT treatment targeting cancer surface cell antigens yielded a significantly longer survival time compared to untreated control mice.

**FIGURE 4:**
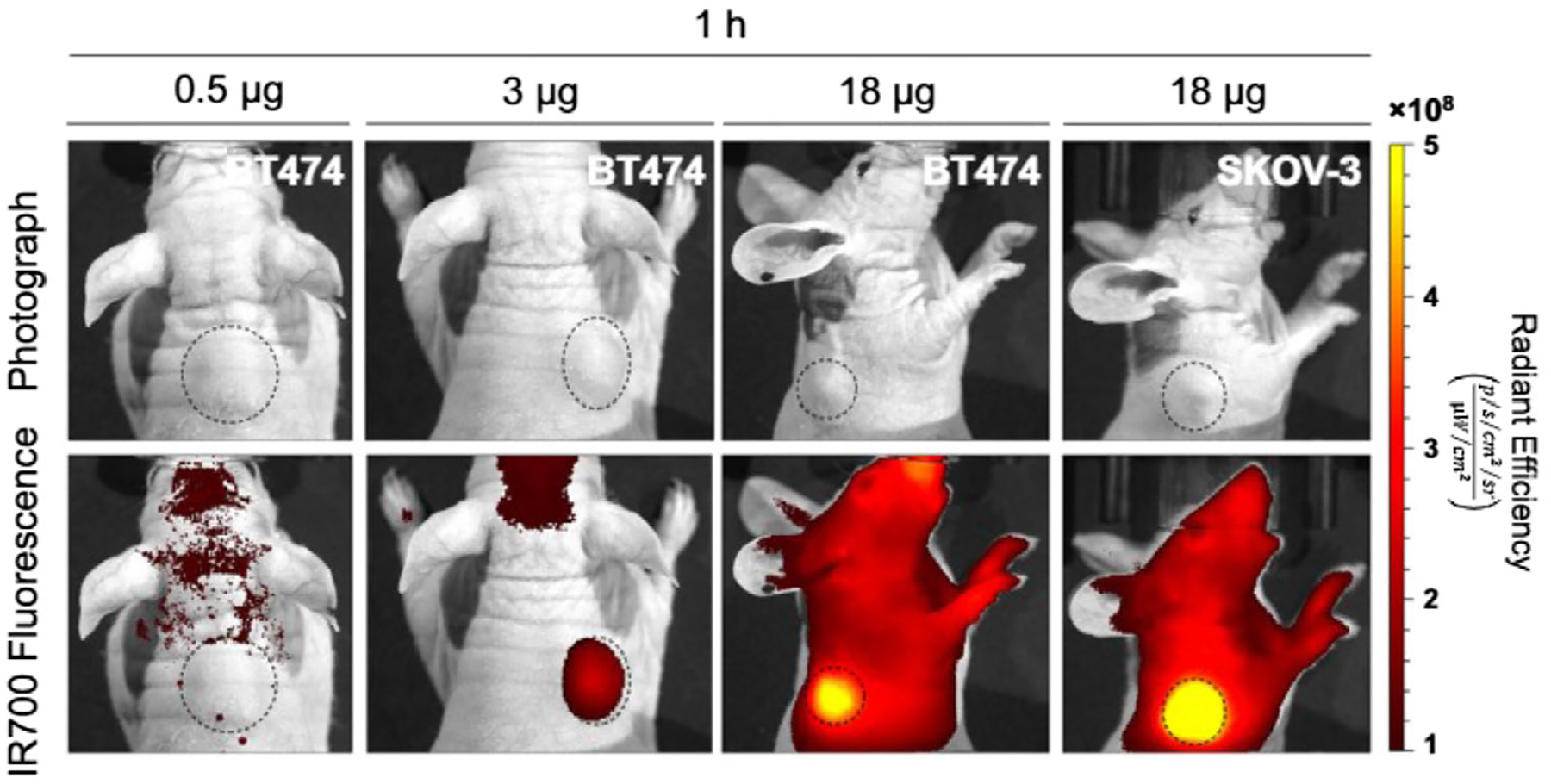
Fluorescence imaging upon NIR-PIT. Fluorescence images of mice bearing HER2 ^+^ xenografts acquired 1 h after injecting affibody Z_HER2:2395_-IR700 conjugate. Reprinted from Maczynska et al.^66^ 2020 with permission from the Nature Publishing Group

The targeting of human epidermal growth factor receptor 2 (HER2), a membrane tyrosine kinase that is commonly overexpressed in solid tumors and is closely related to EGFR signaling,^64^ has many studies underway due to the recent successes of EFGR-targeted NIR-PIT in clinical studies in head and neck cancer.^41^ In one recent study, the HER2-targeted affibody, which is a small engineered peptide that imitates monoclonal antibody activity with high affinity, Z_HER2:2395_, was conjugated to IR700 and applied to SKOV-3 tumor cells followed by NIR exposure, which led to significant HER2-positive cell death, maturation of DCs, and activation of subsequent anticancer immune responses via release of DAMPs in an in vitro model.^66^ In vivo testing results showed visualization of fluorescence in mouse model of xenograft tumors as soon as 1 h after injection of Z_HER2:2395_-IR700, and consistently demonstrated that tumor growth was inhibited successfully to prolong survival of mice compared to untreated control group tumor mice upon NIR irradiation (Figure 4). These encouraging in vitro and in vivo results from NIR-PIT against tumor biomarkers in preclinical models of lymphoma, bladder cancer, and EGFR^+^ or HER2^+^ solid tumors^67^ lay a foundation for continued studies of NIR-PIT targeting various tumor antigens including podoplanin (PDPN),^68^ delta-like protein 3 (DLL3),^69^ and prostate-specific membrane antigen (PSMA)^70^ (Table 1).

## 6 | NIR-PIT TARGETING IMMUNOSUPPRESSIVE POPULATIONS IN ANIMAL MODELS

Along with targeting cancer surface biomarkers, NIR-PIT can also be used to target nontumor cells to augment anticancer responses. CD25 receptors are expressed on the surface of Tregs, which is a key factor associated with immunosuppression of effector T and NK cells at the tumor site. Therefore, targeted therapy for CD25 holds the potential to reverse the suppression of anticancer immunity by blocking Treg activity.^71^ In studies where CD25 was targeted alone, Tregs along with other immune and nonimmune populations were depleted due to shared expressions of surface CD25, which may ultimately weaken the immune response.^72^ In a preclinical study, CD25^+^CD4^+^FOXP3^+^ regulatory T cells (Tregs) were more specifically targeted, which are found naturally within the human immune system, and are integral in self-tolerance and homeostasis, but also contribute to suppressing antitumor immune response.^73^ In preclinical studies of NIR-PIT targeting CD25^+^CD4^+^FOXP3^+^ Tregs, long-term restoration of local antitumor immunity and remission in tumors was established upon combination with cytokine therapy, indicating more specific cancer-expressing CD25 targeting.^74^ Another method to overcome general CD25-targeted cell depletion was a combination approach to target both CD44 and CD25 to selectively bind to cancer cells. CD44 is a gene expressed on cancer stem cells, which is linked to resistance to apoptosis and uncontrolled growth and promotes cancer progression.^75^ NIR-PIT targeting CD44 when combined with the immune checkpoint blockade using anti-PD-1 or CTLA4 antibody, or cytokine (IL-15) administration proved to be effective.^76^ Consistently, the combined CD44 and CD25-targeted therapy proved most effective for colon cancer expressing luciferase (MC38-luc) with high expression of CD44. Comparably, it was less effective for murine oral carcinoma (MOC1) with low expression, the control groups, CD44 target-only groups, and CD25 target-only groups in a syngeneic mouse model,^77^ justifying the efficacy of the dual targeting strategy.

Similarly to targeting Tregs, NIR-PIT can also target other components in the TME, such as cancer-associated fibroblasts (CAFs), which directly relates to the survival of tumor cells. CAFs play an important role in the proliferation and therapeutic resistance of tumor stroma to conventional cancer treatments, which denotes that CAFs are an integral component for cancer cell survival in the host.^78^ In a recent study, Watanabe et al., demonstrated that targeting CAFs with NIR-PIT inhibited esophageal cancer (EC) growth in a xenograft model without adverse effects.^79^ EC is a notoriously aggressive malignant cancer and exhibits less than satisfactory outcomes in terms of treatment success when treated with conventional chemotherapy.^80^ In response to this, Katsube et al. further demonstrated that targeting CAFs with NIR-PIT suppressed the cancer’s resistance to chemotherapy, which could be a viable combination treatment for EC in a xenograft model.^81^ In this study, IR700 was conjugated to a fibroblast activation protein (FAP)-specific antibody to target CAFs. In vitro assay confirmed that the presence of CAFs in EC cells promoted resistance to the conventional therapies of chemotherapy and radiotherapy. In vivo, CAF elimination by NIR-PIT demonstrated that the combination of 5-FU and NIR-PIT produced 70.9% tumor reduction, while 5-FU alone achieved only 13.3% reduction, suggesting that the elimination of CAFs recovered sensitivity of CAF-rich tumors to chemotherapy.

Nishimura et al. targeted tumor microvasculature using antivascular endothelial growth factor receptor 2 (VEGFR-2) mAb, DC101 conjugated to IR700.^82^ In this study, NIR-PIT did not show phototoxicity on NCI-N87 human gastric cancer cells in vitro because of the absence of expression of VEGFR-2 in these cancer cells. However, NIR-PIT targeting VEGFR-2 induced tumor growth delay in NCI-N87 xenografts with damage in tumor vasculature. These studies warrant further study of new methods for combination NIR-PIT, such as simultaneously targeting the TME including CAFs and angiogenic vessels, along with tumor biomarkers to explore a new therapeutic strategy for drug-resistant cancer.

## 7 | ADVANCED LASER DELIVERY METHODS FOR NIR-PIT IN ANIMAL MODELS

Current and traditional NIR-PIT requires external NIR light irradiation at the local area of the tumor to activate the mAb-PS. A drawback to external NIR irradiation is that it sometimes cannot reach far enough to target deep-tissue tumors. To overcome this limitation, an elaborate delivery method has been studied in animal models. A catheter with NIR light emitting diodes (LEDs) has been implemented in NIR-PIT by insertion in the biliary duct to treat cholangiocarcinoma (CCA), a rare and aggressive type of deep-tissue gastrointestinal cancer, in xenograft cancer models in immunodeficient mice.^83^ This study is based on the previous success of reports of endoscopic delivery of NIR irradiation using a fiber optic diffuser to reach deep-tissue gastric cancer in mice.^84^ The advantages of using a catheter to treat deep-tissue tumors using NIR-PIT is that they are flexible and malleable to move about narrow ducts, allowing for deep insertion and radiation. In this study, NIR-PIT was designed to target overexpressed growth factor receptors, EGFR and HER2 in CCA cells, using mAbs panitumumab and trastuzumab, respectively, both conjugated to IR700. CCA-tumor mouse models were used to compare the effects of panitumumab-IR700 and NIR light through methods using the catheter, external NIR light, and a no-light control. Results showed that tumor growth was significantly suppressed in mouse models that were injected with panitumumab-IR700 and exposed to NIR light, either through the catheter or externally, compared to mouse models that were only exposed to one or neither of these factors, confirming that internal irradiation using a catheter is an efficient method of NIR-light delivery to tumors in deep tissue. This early endoscopic study of NIR-PIT warrants further study of deep-tissue tumor treatment feasibility in clinical settings, and future clinical trials.

## 8 | NIR-PIT CLINICAL STUDIES

**FIGURE 5:**
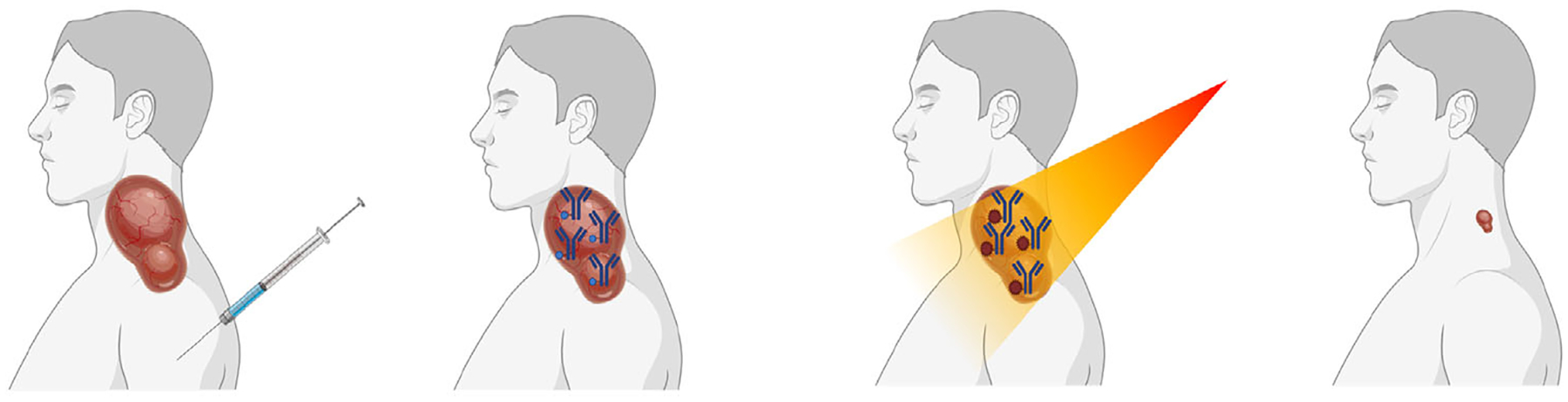
Clinical study of NIR-PIT on head and neck cancer. After subcutaneous injection of cetuximab–IR700 complex targeting EGFR, followed by NIR light treatment on the head and neck cancer resulted in subsequent tumor shrinkage and anticancer immune responses

Clinical trials of NIR-PIT have been in Phase 1/2/3 globally since 2015 (Table 2). The first clinical study of NIR-PIT was completed in 2019 using the target antibody cetuximab saratolacan (cetuximab conjugated to IR700, also known as RM-1929) to target EFGR in patients with recurrent head and neck cancer to demonstrate its safety and efficacy (NCT02422979).^85,86^ Head and neck squamous cell carcinoma is located at the base of the head where it connects to the neck, and is the sixth most common cancer globally.^87^ This approach involves subcutaneous injection of the cetuximab-IR700 complex targeting EGFR and subsequently shining NIR light onto the local skin area of the head and neck (Figure 5). This approach was further used in a Phase 2a trial.^85^ Results indicated objective response rate (ORR) of 50% (15/30), complete response (CR) of 16.7% (5/30), and disease control rate (DCR) of 86.7% (26/30).

Success in reduction of tumor burden and infrequency in cancer recurrence in this trial sparked more clinical trials to be approved and progress forward, which are still ongoing.^41^ One ongoing Phase 3 clinical trial, which began in 2019, employs the antibody–dye conjugate of cetuximab and IR700 targeting EGFR, ASP-1929, in patients with recurrent head and neck cancer who have failed attempted conventional cancer treatment more than two times previously in three countries in Asia, the US/Canada, and four countries in the EU (NCT03769506).^88^ This trial will record OS and PFS in patients. Another new clinical trial, which began in December 2020, is attempting to treat multiple types of metastatic squamous cell carcinoma that express EGFR, via combination treatment of the same antibody–dye conjugate, ASP-1929, in conjunction with anti-PD1 checkpoint therapy (NCT04305795).^86^ This trial will record OS, PFS, and duration of response (DOR) in patients. The results of these ongoing clinical trials will be pertinent to the continued research and development of NIR-PIT.

## 9 | FUTURE DIRECTIONS

### 9.1 | Combination therapy

NIR-PIT gives hope for new combination cancer treatments implementing chemotherapy, radiotherapy, surgery, or other phototherapies to achieve the most successful outcomes in both cytotoxic effect and augmentation of anticancer immune response. In a study combining NIR-PIT with a chemotherapy drug, trastuzumab-IR700 was bound to the highly potent cytotoxic drug, maytansinoid DM1, and targeted HER2 cells in both large and small xenograft tumor models.^89^ DM1 is a chemotherapy agent that releases catabolites to bind to tubulin upon activation, resulting in mitotic arrest, inhibition of cell growth, and cell death. This chemotherapy–NIR-PIT combination treatment resulted in reduced tumor volume and prolonged survival in large tumor models that could not receive sufficient NIR exposure compared to tumor models that were only treated with trastuzumab-IR700 alone. This approach could be used to effectively treat tumors that are difficult to expose to therapeutic NIR light upon NIR-PIT.

In a study that investigated the effects of combination trastuzumab HER2-targeted therapy with adjuvant chemotherapy, 1 year of trastuzumab treatment significantly increased disease-free survival in breast cancer patients following the completion of primary therapy compared with no treatment groups.^90^ Along with this result, trastuzumab therapy allowed for extended immune recruitment at the tumor site compared to control groups, which opened the door for subsequent Her2-NIR-PIT that would enhance the antitumor immunity effect.

### 9.2 | Drug delivery in hyperpermeability induced by NIR-PIT

**FIGURE 6:**
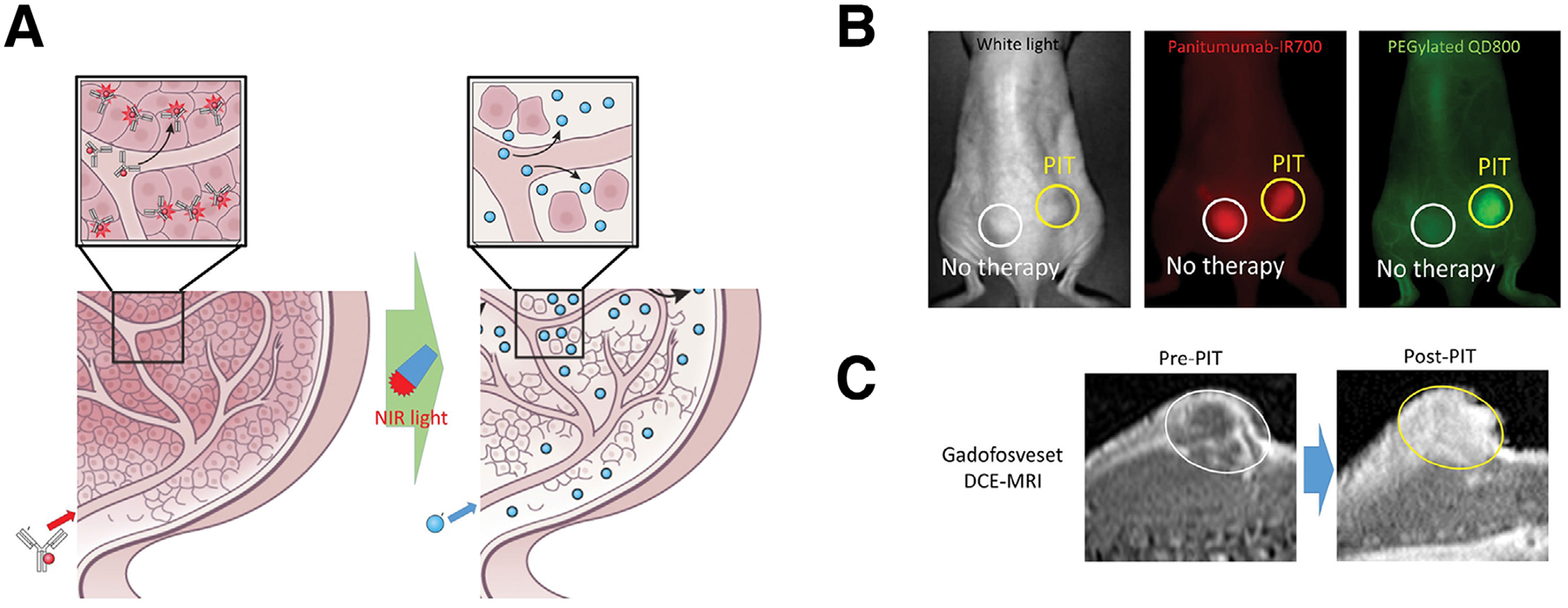
Mechanisms of action of super-enhanced permeability and retention (SUPR) upon NIR-PIT. (A) Selective killing of the tumor in the perivascular space causes dilation in the nearby tumor vessels and subsequently causes an increase in microvascular permeability, which is termed as SUPR. This effect could enhance delivery of various nano-sized agents into tumor beds. (B) SUPR enhanced delivery of antibody conjugates (red) and quantum dots (green) into NIR-PIT treated tumor after intravenous injection. (C) SUPR enhanced delivery of gadofosveset, an MRI contrast agent. (B and C) Circles indicate tumors. Reprinted from Kobayashi et al.^93^ 2016 with permission from the Royal Society of Chemistry

The tumor vasculature features enhanced permeability and retention (EPR) effects, due to its leaky endothelial barrier and poor access to lymphatic drainage.^91^ EPR allows for nanosized drugs to extravasate preferentially into tumor tissue through the permeable tumor vessels and increases the delivery of small particles to the tumor bed, which naturally makes tumor cells more targeted by nanotherapeutics than normal cells in the body. However, EPR often provides insufficient impact in providing effective cancer treatment.^92^ Interestingly, NIR-PIT uniquely induces a super-enhanced permeability and retention (SUPR) effect by selectively killing the tumor in the perivascular space and causing dilation in the tumor vessels (Figure 6).^93^ Enlargement of the vessel leads to significantly increased nanodrug leakage into the tumor bed and homogeneity of drug spread. Visualized through fluorescence imaging and MRI, recent studies were performed in which targeted quantum dots, nanoparticles, and contrast agents mimicking cancer nanodrugs rapidly accumulated in tumor tissues treated with NIR-PIT.^93,94^ Further research is needed to fully understand the impact of NIR-PIT on cancer nanodrug delivery, but the SUPR effect provides a promising opportunity for combination therapy implementing current and candidate nanodrugs.

### 9.3 | NIR-PIT implementation as an imaging modality

**FIGURE 7:**
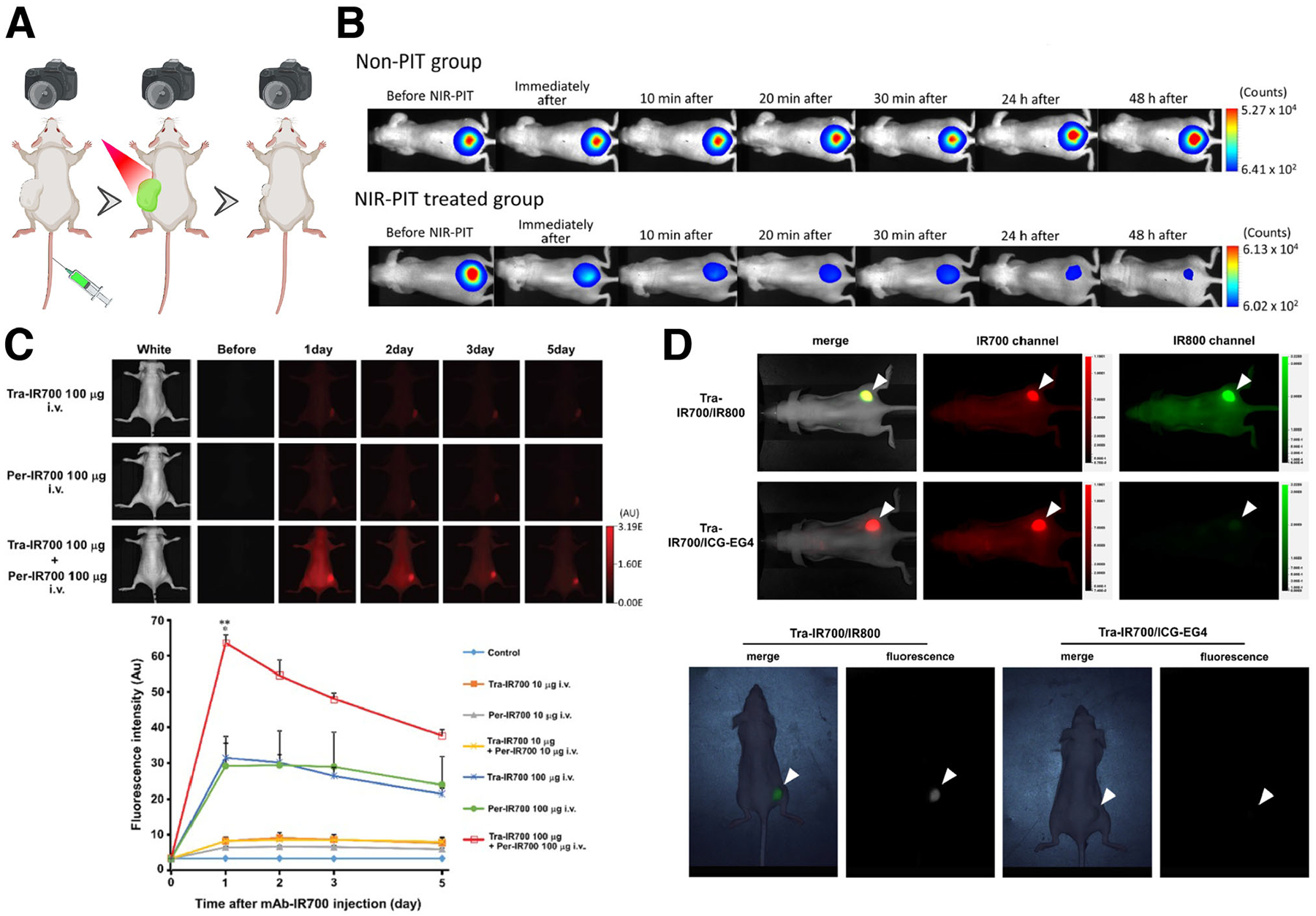
Diagnostic imaging with NIR-PIT. (A) Schematic for fluorescence imaging with NIR-PIT. NIR imaging could be used for real-time detection of tumors, without damaging the tumor tissues, upon NIR-PIT. (B) Luciferase-luciferin photon-counting real-time images of A431-luc-GFP tumor bearing mice for NIR-PIT. Luciferase activity continued to decrease after NIR-PIT, showing the loss of viability in treated cancer cells in the lower panels compared to untreated cells in the upper panels. Reprinted from Maruoka et al.^95^ 2017 with permission from the Royal Society of Chemistry. (C) Time course determination of biodistribution of trastuzumab-IR700 and pertuzumab-IR700 using fluorescence imaging. Upper: NCI-N87 tumor xenografts were visualized with IR700 fluorescence after intravenous injection of the antibody conjugates. Lower: IR700 signals reached their peak 1 day after co-injection of the antibody conjugates. Reprinted from Ito et al.^100^ 2016 with permission from the Impact Journals, LLC. (D) In vivo imaging of antibody conjugates of trastuzumab-IR700/IR800 and trastuzumab-IR700/ICG-EG4, which have an additional conjugation of IRDye800CW (IR800) or ICG-EG4-Sulfo-OSu (ICG-EG4) to trastuzumab-IR700. Upper: Fluorescence images of trastuzumab-IR700/IR800 and trastuzumab-IR700/ICG-EG4 in tumor-bearing mice obtained with the Pearl Imager. Lower: Fluorescence images of trastuzumab-IR700/IR800 and trastuzumab-IR700/ICG-EG4 in tumor-bearing mouse obtained by LIGHTVISION. In these imaging systems, the fluorescence of IR800 was clearly detected, suggesting that this fluorophore can be used to determine biodistribution of the conjugate. Reprinted from Inagaki et al.^99^ 2021 with permission from the American Chemical Society

Imaging methods for pre- and post-NIR-PIT treatment are useful to assess the immediate and long-term effects of NIR-PIT to the TME, such as necrotic and immunogenic changes (Figure 7). In a study comparing IR700 and GFP fluorescence imaging and luciferase-luciferin photon-counting to measure the therapeutic effects of NIR-PIT, athymic nude mice were injected with A431-luc-GFP cells to express human epidermal growth factor receptor 1 (HER1)-tumors and genes encoding luciferase and GFP.^95^ The mice were treated with panitumumab conjugated with IR700 (pan-IR700), targeting HER1, and irradiated with 50 J/cm^2^ of NIR light, in which luciferase-luciferin photon-counting and fluorescence imaging were compared up to 2 days after NIR-PIT treatment to visualize therapeutic response in tumors. NIR fluorescence from pan-IR700 was lower after NIR-PIT in the PIT-treated group than in the non-PIT group, confirming the successful therapeutic response to NIR light irradiation (Figure 7B). Tumor volumes in the group treated with NIR-PIT were significantly less and survival was prolonged compared to the untreated control group, which indicated low viability of tumor cells and confirmed that the NIR-PIT treatment was successful. Luciferase-luciferin photo-counting imaging resulted in the best representation of tumor viability in both the early and late stages, because intensity reductions were more sensitive and synchronous with actual tumor NIR-PIT response. The use of NIR fluorescence and luciferase bioluminescence imaging was also successful in another NIR-PIT study targeting CD47 in human bladder cancer by transfecting 639V cell lines with GFP-luciferase in mouse xenograft models.^96^ In this study, NIR fluorescence reached its peak 1 day after injection, indicating that the max. accumulation of anti-CD47 antibody conjugated with IR700 in tumor cells was achieved in 24 h after injection. After NIR irradiation at the 24 h time point, an increase in luciferase-luciferin photo-counting was much less than that in non-irradiated controls, indicating that NIR-PIT successfully decreased viability of tumors. A concern regarding luciferase-luciferin photo-counting imaging is that it is not translatable for clinical use, therefore this imaging strategy for NIR-PIT requires further research and development.

NIR-PIT methods can also potentially be used for diagnostic imaging. Using the clinically approved ICG imaging modality, LIGHTVISION, tumor detection was accomplished using IR700 fluorescent dye irradiated by low-power 690-nm laser light. ICG operates within a wide window of the NIR spectrum, between 700 and 900 nm, which allows ICG imaging modalities to also pick up on signals near this range, such as IR700. IR700 is excited by NIR light at a wavelength of 690 nm, and emits a peak at 702 nm, also exhibiting a long tail of fluorescence that extends toward 800 nm.^97^

In conventional NIR-PIT, irradiation of the APC by 690 nm NIR light is used to elicit rapid cell death in tumor cells and stimulate host immune response. Inagaki et al. demonstrated that the same 690 nm NIR light for NIR-PIT could be used for real-time detection of tumors, without damaging the tumor tissues, when operated at a lower power.^98^ Irradiation between 0.5 and 2.0 mW/cm^2^ output power had no therapeutic effect or fluorescence decay indications on the tumor cells, which suggests that there is a duality in the function of NIR-PIT: the selective killing of target cells *and* intratreatment tumor detection. The same group further demonstrate that additional conjugation of a fluorophore of IRDye800CW (IR800), which has an overlapping spectrum with ICG, to trastuzumab-IR700 conjugates could allow visualization of target tumors with Pearl Imager and LIGHTVISION (Figure 7D).^99^ These findings warrant further study in IR700 conjugated to specific antibodies as real-time tumor-detecting agents for cancer diagnosis, and offers the opportunity to develop imaging modalities other than LIGHTVISION to yield maximum results. If conjugated IR700 can be reliably and safely used as a tumor-detecting agent at low-power NIR irradiation, then it can be used as a preliminary biopsy to assess cancer burden and accumulation of a targeted conjugate and can subsequently monitor biodistribution of the conjugate real-time during NIR-PIT treatment.

Fluorescence imaging with NIR-PIT was also used in a study implementing the combination treatment with HER2-targeting trastuzumab-IR700 (Tra-IR700) and pertuzumab-IR700 (Per-IR700).^100^ Combination treatment of both agents was compared to using one of the agents alone to measure differences in fluorescence intensity upon fluorescence imaging. HER2-expressing NCI-N87 cells and NIH/3T3 control cells were stained with Tra-Alexa488 and Per-IR700 fluorescence dyes that both accumulated predominantly in NCI-N87 cells but were not visualized in NIH/3T3 cells, which showed that Tra-Alexa488 and Per-IR700 specifically binds to HER2-expressing tumor cells. In vitro cytotoxicity results showed that the combination of Tra-IR700 and Per-IR700 with NIR light irradiation yielded the greatest rate in cell death, compared to one of the agents alone with NIR light. In NCI-N87 xenograft tumor models, the mAb-IR700s were injected intravenously and exposed to IR700 fluorescence. The fluorescence intensity of IR700 between 1 and 5 days postinjection was associated with the treatment effect. The combination treatment of both Tra-IR700 and Per-IR700 with NIR light led to the strongest fluorescence intensity and antitumor effect compared to control groups (Figure 7C). This study showed that combination treatment of targeted photosensitizing agents may be more effective than single-agent treatments, and that the monitoring of tissue accumulation of targeted photosensitizing agents could predict a treatment outcome through fluorescence imaging. These results warrant further studies on the use of imaging modalities to determine tumor microdistribution of mAb-PSs and assess the treatment outcomes.

### 9.4 | PIT using small molecules

**FIGURE 8:**
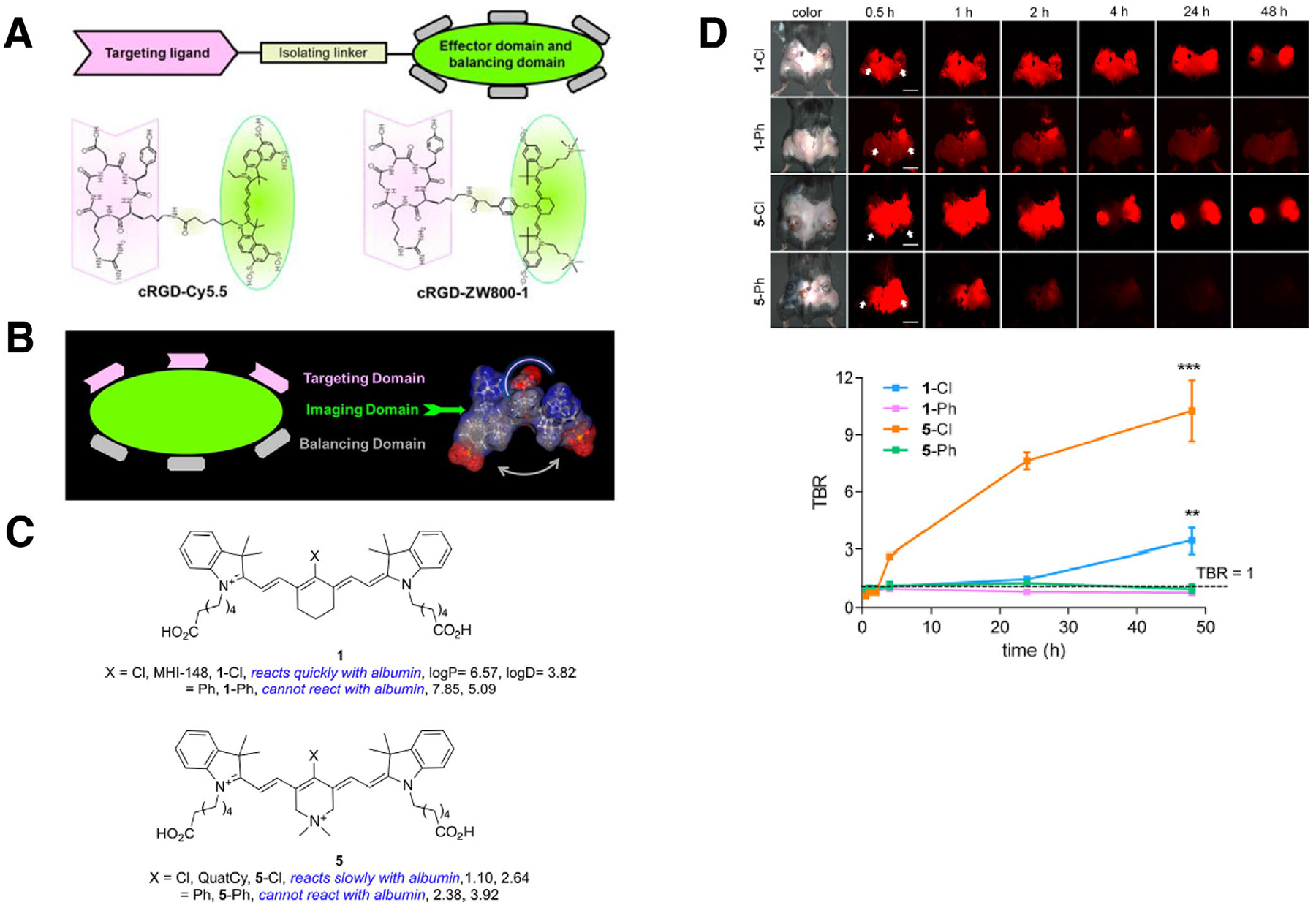
Structure-inherent targeting. (A) Modular design for targeted conjugates of cRGD-conjugated Cy5.5 and ZW800–1. (B) Structure-inherent targeting through the incorporation of targeting, imaging, and balancing domains into a small molecule. (A and B) Reprinted from Owens et al.^102^ with permission from the American Chemical Society. (C) Tumor-seeking “QuatCy” and four related polymethine compounds: 1-Cl, 1-Ph, 5-Cl (QuatCy), and 5-Ph. 1-Cl or 5-Cl reacts under physiological conditions with serum albumin via displacement of the meso chloride, while 1-Ph or 5-Ph does not. (D) Tumor targeting of 1-Cl, 1-Ph, 5-Cl, and 5-Ph. Time course of tumor to background ratios (TBRs) is shown. Note that 5-Cl (QuatCy) with albumin binding capability showed strong tumor targetability. Reprinted from Usama et al.^113^ 2020 with permission from the American Chemical Society

Thus far, PIT has been performed using an NIR dye conjugated to an antibody or affibody for tumor targeting. A number of new probes based on nanoparticles, antibodies, proteins, peptides, or small molecules have been developed to achieve target-specific binding.^101,102^ However, multifunctional nanoparticles often showed undesired accumulation in vital organs and raised safety concerns including unknown long-term toxicity.^103^ Anti-bodiestargeting biomarkers on cancer cells are generally too large to penetrate deeply into tumor tissue and showed slow clearance, which caused nonspecific accumulation in the excretory organs^104^ and prolonged waiting time (often up to several days) after administration to achieve target-specific accumulation,^103^ making this approach less attractive. In future PIT studies, it would be worthwhile to explore the possibility of using smaller molecules to improve the efficacy of PIT and expand its applicability to a wider range of cancer types. Small molecules measuring 10–1000-fold smaller than peptides and proteins can rapidly distribute in their targets, and unbound molecules can be rapidly excreted^105^ avoiding nonspecific accumulation in off-target tissues. This goal could be achieved by using a smaller ligand of cyclic arginine-glycine-aspartate motif (cRGD) or folate analog targeting high expression of integrin *α*_V_ or the folate receptor *α* (FR*α*) in tumor tissue, respectively (Figure 8A). Clinical data showed that these approaches exhibit rapid renal clearance after intravenous injection and typically require an injection 2–18 h prior to surgery.^106–108^ In particular, a recent Phase II study showed that OTL38, which is a folate-indole-cyanine green-like conjugate targeting FR*α*, was safe and could intraoperatively identify more ovarian cancer lesions during surgery than traditional detection techniques alone.^106^ However, due to the modest expression of integrin *α*_V_ or FR*α* in normal tissue,^109^ these approaches resulted in a high false positive rate in clinical testing.^107,110^ In addition, these approaches still rely on the conventional bioconjugation, which is a complicated step and hampers their clinical translation due to difficulties in economical, large-scale, and reproducible production. Together, these suggest that such conventional approaches require significant improvement for clinical molecular imaging.

Both PDT and PTT have reached stages in which smaller probes can be employed to achieve tumor-targeting and induce cytotoxicity, which alludes to the feasibility of NIR-PIT also implementing small molecules in targeting specific cancer cells. Besides porphyrins, in various PDT studies, boron dipyrromethene (BODIPY) fluorophores were engineered into PSs and proved successful as both a dual-imaging and therapeutic agent in vivo and in vitro.^111^ In terms of PTT, as mentioned earlier in this review, the organic fluorophore ICG is commonly used. For a small molecule to be successful in NIR-PIT, it would have to be engineered to specifically target tumor tissue and induce necrosis in cells when exposed to NIR light. This goal can be achieved by developing ultrasmall fluorescence probes that exploit chemical recognition to biological tissue and show specific targeting for tumor or normal tissues.^102^ In this “structure-inherent targeting,” all the necessary components that are commonly achieved by bioconjugation (Figure 8A) are all integrated into a small molecule (Figure 8B). The currently approved small NIR fluorescence probe–ICG–with a molecular weight of 774.96, has shown significant limitations with low sensitivity and specificity, and higher liver and gastrointestinal tract uptake due to the nontargeted nature and undesirable biodistribution of the molecule.^110^ Contrary to this, structure-inherent targeting probes are tuned for the lipophilicity and noncharged structures, which allows for high uptake in the specific tissue such as endocrine system, bone, and nerve.^112^ For example, recent research has reported that in the class of cyanine-based “tumor-seeking” dyes, a central chloride on the molecule’s heptamethine bridge is integral in forming covalent adducts with albumin in cancer cells, which allows enhanced, specific, and prolonged retention of the dyes in tumor cells (Figure 8C,D).^113^ Further studies of this approach could provide useful insight into an effective mechanism for small-molecule targeting in NIR-PIT, PDT, or PTT. The use of small molecules represents many advantages over the conventional approaches that need bioconjugation; it is a single small molecule rapidly distributed to target tissues and eliminated from the body to avoid nonspecific uptake in off-target tissues. Importantly, it also allows for large-scale, rapid, and reproducible production with reasonable cost for clinical use. The therapeutic effect relies on accumulation and retention of the therapeutics at the target tissue. To this end, (a) passive targeting via the EPR effect and (b) active targeting using the innate biodistribution of known molecules can be employed.^102,114^ Passive targeting via the EPR effect typically favors a relatively large molecule in diameter.^114^ Surface biomarkers in tumor cells have been used for homing small-molecule therapeutics directly to cancerous tissues. While this is a rapid process that offers the specific, rapid elimination of small molecules from the system, it negatively affects retention in the target tissue. With its fast clearance, small molecules often require higher dosage injection to achieve sufficient accumulation in the targeted tumor tissue for therapeutic purpose.^102,114,115^ For optimal targeting, specificity, delivery, pharmacokinetics, and toxicity, the chemical structure of the small molecule can be rationally designed by the addition or removal of certain functional groups.^102^ Specific for NIR fluorophores, such designs often face constraints to preserve the fluorescence domain within its structure.^102,103^ Future studies are warranted to overcome these unique challenges in the use of small molecules and determine if necrosis in tumor cells triggered by a targeted small molecule could induce robust immune response upon photomedicine.

## 10 | CONCLUDING REMARKS

Today, there are many types of cancer that continue to only have the limited treatment options of surgery, chemotherapy, and radiation, which leaves overall prognosis for these cancers poor, especially at advanced stages. Photomedicine, in particular, PIT holds the potential to overcome these limitations through its unique and specific mechanism. The most unique feature of NIR-PIT is its ability to induce ICD via stress responses and necrosis in targeted cancer cells upon NIR light exposure. ICD is a crucial contributor in initiating the rapid maturation of DCs and subsequent priming of T cells, which provides the host with long-lasting antitumor immunity. The prospect of combining the immunogenic effects of ICD with other conventional and candidate cancer treatment methods provides a promising approach to both directly killing primary cancer cells and re-activating the suppressed immune system to prolong disease-free survival and survival rates in cancer patients.

## Figures and Tables

**TABLE 1:** Preclinical studies of NIR-PIT

Target molecule	Tumor	Targeting agent	Cell line	Treatment outcome	Immunological outcome	References
CLA	Mycosis fungoides	Anti-CLA-IR700	My-La CD4+	Tumor cell death (in vitro)	N/A	^63^
HER2 EGFR	Epidermoid squamous carcinoma	Trastuzumab-IR700, cetuximab-IR700	3T3-HER2, A431	Tumor cell death, tumor cell swelling (in vitro)	DAMPs and tumor antigen release, DCs maturation (in vitro)	^58^
CD47	Bladder cancer	Anti-CD47-IR700	UMUC3, 639V, and HT1376	Tumor growth inhibition	Increased phagocytosis	^65^
EGFR	Bladder cancer	Cetuximab-IR700	TCC, SW780	Tumor growth inhibition	N/A	^67^
HER2	Ovarian cancer Bladder cancer	Trastuzumab-IR700	SKOV-luc-D3, SW780	Tumor growth inhibition	N/A	^67^
HER2	Ovarian cancer Breast cancer	HER2-specific affibody-IR700	SKOV3, BT474	Tumor growth inhibition	Maturation of DCs and activation of subsequent anticancer immune responses via DAMPs	^66^
PDPN	MPM	NZ-1-IR700	MSTO-211H, H2373	Tumor growth inhibition	N/A	^68^
DLL3	SCLC	Rovalpituzumab-IR700	SBC3 and SBC5	Tumor growth inhibition	N/A	^69^
PSMA	Prostate cancer	Anti-PSMA-IR700	PC3-PSMA+ (PC3pip), PC3-PSMA-(PC3flu)	Tumor growth inhibition	N/A	^70^
HER2	Gastric cancer	Trastuzumab-IR700	N87GFP	Tumor growth inhibition using a fiber optic diffuser to reach deep-tissue gastric cancer	N/A	^84^
HER2 EGFR	Cholangiocarcinoma	Trastuzumab-IR700, cetuximab-IR700	HuCCT-1	Tumor growth inhibition using a fiber optic diffuser to reach deep-tissue cholangiocarcinoma	N/A	^83^
VEGFR-2	Tumor vasculature in gastric cancer	DC101-IR700	NCI-N87	Tumor growth inhibition	N/A	^82^
FAP	CAFs in esophageal squamous cell carcinoma	Anti-FAP-IR700	TE4, TE8, FEF3	Tumor growth inhibition Enhanced tumor growth inhibition with combination of 5-FU and NIR-PIT	N/A	^79,81^
CD44	Colon, lung, oral cancer	Anti-CD44-IR700	MC38-luc, LLC, MOC1	Tumor growth inhibition	Combination of NIR-PIT with PD-1 or CTLA4 blockade, or IL5 administration combination induced growth inhibition of distant tumors and increased CD8 T-cell infiltration in tumors	^76^
CD25	T regs in lung, breast, colon cancer	Anti-CD25-IR700	LL/2, 4T1, MC38	Tumor growth inhibition Enhanced tumor growth inhibition with IL-15 administration	Enhanced IFN-*γ* expression in CD8^+^ T cells and NK cells	^74^
CD44 and CD25	Tregs and cancer stem cells in colon, lung, oral cancer	Anti-CD44-IR700, anti-CD25-IR700	MC38-luc, LL/2, and MOC1.	Tumor growth inhibition	Increased T-cell infiltration into the TME	^77^
HER2	Fibroblast	Trastuzumab-IR700-DM1	NIH 3T3/HER2	Tumor growth inhibition Enhanced efficacy of the combinational therapy of NIR-PIT and chemoimmunotherapy	N/A	^89^
EGFR	Breast	Panitumumab-IR700-duocarmycin	MDAMB468-luc	Tumor growth inhibition Additional greater permeability and penetration (SUPR effects)	N/A	^94^
HER1	Epidermal carcinoma	Panitumumab-IR700	A431-luc-GFP cells	Tumor growth inhibition Loss of fluorescence of the targeted agent after NIR irradiation Dual imaging of cell viability and targeting of the agent	N/A	^95^
CD47	Ureter urothelial carcinoma	Anti-CD47 antibody-IR700	639V cells	Tumor growth inhibition Loss of fluorescence of the targeted agent after NIR irradiation Dual imaging of cell viability and targeting of the targeting agent	Macrophage infiltration into tumor	^96^
HER2	Gastric cancer	Trastuzumab-IR700	NIH-N87-GFP/luc	Tumor growth inhibition LIGHTVISION imaging of biodistribution of the targeting agent	N/A	^98^
HER2	Gastric cancer	Trastuzumab-IR700/IR800	NIH-N87-GFP/luc	Tumor growth inhibition Pearl Imager and LIGHTVISION imaging of biodistribution of the targeting agent	N/A	^99^
HER2 EGFR	Gastric cancer	Trastuzumab-IR700, pertuzumab-IR700	NCI-N87 cells NIH/3T3	Tumor growth inhibition correlated with positive biodistribution of the targeted agents	N/A	^100^

*Note*: Preclinical studies of NIR-PIT to date with detailed pieces of information on tumor models, targeted construct used, treatment, and immunological outcomes are listed.

Abbreviations: CAFs, cancer-associated fibroblasts; CLA, cutaneous lymphocyte antigen; DLL3, delta-like protein 3; EGFR, epidermal growth factor receptor; FAP, fibroblast activation protein; MPM, malignant pleural mesothelioma; NIR-PIT, near-infrared photoimmunotherapy; PDPN, podoplanin; PSMA, prostate-specificmembrane antigen; SCLC, small-cell lung cancer; TCC, transitional cell carcinoma; TME, tumor microenvironment; VEGFR-2, vascular endothelial growth factor receptor 2.

**TABLE 2:** Clinical studies of NIR-PIT

Number	Year	Statue	Type	Phase	Number of patients	Patient population	Agents used	Clinical outcome	Ref.
NCT02422979	2015	Completed	Intervention	Phase 1	40	Recurrent head and neck cancer	RM-1929	Tumor density was decreased in 7/8, with ORR 75% and DCR100%	^86^
NCT02422979	2015	Completed	Intervention	Phase 2	30	Cannot be satisfactorily treated with surgery, radiation, or chemotherapy	RM-1929	ORR was 50*%* (15/30), with 16.7% (5/30) CR and 86.7% (26/30) DCR	^85,86^
NCT04305795	2020	Ongoing	Intervention	Phase 1/2	74	Recurrent or metastatic HNSCC or advanced or metastatic cutaneous squamous cell carcinoma	ASP-1929 + anti-PD-Ll	TEAE and serious TEAE, OS, PSF, DOR	^86^
NCT03769506	2018	Ongoing	Intervention	Phase 3	275	Recurrent HNSCC who have failed at least two lines of therapy	ASP-1929	OS, PSF	^86,88^

*Note*: Clinical trials of NIR-PIT to date with detailed pieces of information on trial registrations, patient populations, agents used, and treatment outcomes are listed.

Abbreviations: CR, complete response; DCR, disease control rate; DOR, duration of response; HNSCC, squamous cell cancer of the head and neck; NIR-PIT, near-infrared photoimmunotherapy; ORR, overall response rate; PFS, progression-free survival; TEAE, treatment emergent adverse events.
